# Cytosine Methylation Alteration in Natural Populations of *Leymus chinensis* Induced by Multiple Abiotic Stresses

**DOI:** 10.1371/journal.pone.0055772

**Published:** 2013-02-13

**Authors:** Yingjie Yu, Xuejiao Yang, Huaying Wang, Fengxue Shi, Ying Liu, Jushan Liu, Linfeng Li, Deli Wang, Bao Liu

**Affiliations:** 1 Institute of Grassland Science, Northeast Normal University, Key Laboratory of Vegetation Ecology, Ministry of Education, Changchun, PR China; 2 Key Laboratory of Molecular Epigenetics of Ministry of Education, and Institute of Genetics and Cytology, Northeast Normal University, Changchun, PR China; National Taiwan University, Taiwan

## Abstract

**Background:**

Human activity has a profound effect on the global environment and caused frequent occurrence of climatic fluctuations. To survive, plants need to adapt to the changing environmental conditions through altering their morphological and physiological traits. One known mechanism for phenotypic innovation to be achieved is environment-induced rapid yet inheritable epigenetic changes. Therefore, the use of molecular techniques to address the epigenetic mechanisms underpinning stress adaptation in plants is an important and challenging topic in biological research. In this study, we investigated the impact of warming, nitrogen (N) addition, and warming+nitrogen (N) addition stresses on the cytosine methylation status of *Leymus chinensis* Tzvel. at the population level by using the amplified fragment length polymorphism (AFLP), methylation-sensitive amplified polymorphism (MSAP) and retrotransposon based sequence-specific amplification polymorphism (SSAP) techniques.

**Methodology/Principal Findings:**

Our results showed that, although the percentages of cytosine methylation changes in SSAP are significantly higher than those in MSAP, all the treatment groups showed similar alteration patterns of hypermethylation and hypomethylation. It meant that the abiotic stresses have induced the alterations in cytosine methylation patterns, and the levels of cytosine methylation changes around the transposable element are higher than the other genomic regions. In addition, the identification and analysis of differentially methylated loci (DML) indicated that the abiotic stresses have also caused targeted methylation changes at specific loci and these DML might have contributed to the capability of plants in adaptation to the abiotic stresses.

**Conclusions/Significance:**

Our results demonstrated that abiotic stresses related to global warming and nitrogen deposition readily evoke alterations of cytosine methylation, and which may provide a molecular basis for rapid adaptation by the affected plant populations to the changed environments.

## Introduction

The influences of human activities on global environments have been studied extensively in the past years. It is well documented that human activity has increased the atmospheric concentrations of greenhouse gases which have successively elevated global surface temperatures over the past decades [1]–[3]. The consequences of this temperature changes are more frequent occurrence of extreme weather and climate events leading to global environmental changes. In addition, the global nitrogen cycle has also been altered by human activities such as excessive use of nitrogen fertilizers, legume crops and fossil fuel combustion [4]–[7]. This added nitrogen has a profound effect on the chemistry of the atmosphere, aquatic and terrestrial ecosystems, and ultimately results in changes in global environments and ecosystems. These global environmental changes will result in species distribution shifts, behavioral changes and altered phenology, and these phenomena have been observed in diverse ecological settings [8]. Taken together, these previous studies have demonstrated that human activities caused alterations in global environments and the increase in extreme events under global change will impose episodic stress upon organisms, such as heat, drought and salt [9].

To survive, plants need to continuously adjust their genomes to external stimuli to adapt to the changing and stressful environments [10], [11]. Therefore, the use of molecular techniques to investigate the mechanisms of plant responses to environmental changes has attracted a numerous research efforts. According to the Modern Evolutionary Synthesis, random genetic variations were thought to be the primary sources of heritable adaptation in response to altered environments. Indeed, some studies illustrated that genetic mutations have played a pivotal role in organismal adaptation to various abiotic stress conditions [12]–[17]. In recent years, however, there are increasing numbers of studies addressing how and to what extent the epigenetic alterations might contribute to the plants’ ability to cope with various abiotic stresses [18]–[21]. These studies demonstrated that epigenetic modifications, including DNA methylation, histone modification and RNA interference, could rapidly alter the gene expression levels and chromatin structure, ultimately lead to heritable changes in biochemical, physiological and morphological traits, and some of which play critical roles in response to a particular stress condition [11], [22]–[24]. Nonetheless, the heritability of such induced methylation alterations in plants remain to be fully addressed. Therefore, several recent studies have investigated the transgenerational methylation changes, and documented that the stress-induced alterations in DNA methylation are common and some of these alterations could be stably transmitted to subsequent generations, and which may help plants to adapt to the same or similar stresses their progenitors once experienced [25]–[27].

Despite accumulated studies on how plants sense and adapt to abiotic stresses, the complexity of environmental variations often makes it difficult to distinguish the real underlying causes under laboratory conditions [28], [29]. Therefore, the use of environmentally realistic conditions to address the molecular mechanisms of plants in response and eventual adaptation to abiotic stress is emerging as an important step to evaluate the roles of epigenetic variations. The obvious advantages of such experiments are that the abiotic stresses are much more amenable to be controlled and the interactions between different abiotic stresses can also be assessed.

In this study, we employed the amplified fragment length polymorphism (AFLP), methylation-sensitive amplified polymorphism (MSAP) and retrotransposon based sequence-specific amplification polymorphism (SSAP) techniques to investigate the impact of warming, nitrogen (N) addition and warming+N addition stress on natural populations of a grass species *Leymus chinensis* Tzvel. under the experimental field conditions. Here, we have asked the following questions: (1) To what extent the abiotic stresses like warming and nitrogen addition would affect the cytosine methylation levels and patterns of *L. chinensis*? (2) Would the alterations in cytosine methylation show heterogeneity among different genomic regions?

## Materials and Methods

### Ethics Statement

No specific permits were required for this study, because the performance of this study was in accordance with guidelines set by the Northeast Normal University, China. No specific permits were required for the described field studies, because the field is owned by Northeast Normal University and the Songnen Grassland Ecological Research Station performs the management. No specific permits were required for these locations/activities, because the location is not privately-owned or protected in any way and the field studies did not involve endangered or protected.

### Plant Materials and Experimental Design

The species *L. chinensis* Tzvel., which belongs to the genus *Leymus* of family Gramineae, is a dominant species of grassland ecosystem and widely distributed in the Eurasian Steppes [30]. Previous studies demonstrated that this grass species has highly tolerance to several abiotic stresses, and exhibited remarkable plasticity in the morphological and physiological characteristics [31]. These attributes render *L. chinensis* as an ideal plant to study the molecular mechanisms of adaptation to abiotic stress conditions. In the present study, a field-cultivated experiment of *L. chinensis* was conducted at the Songnen Grassland Ecological Research Station of Northeast Normal University (44°40′ N, 123°44′ E). It has a semi-arid, continental climate with mean annual temperature 4.6–6.4°C and annual precipitation 280–400 mm, and soils are mixed saline and alkaline. The species *L. chinensis* is a clonal perennial grass with large below ground bud bank and could rapid propagate through asexual reproduction. These attributes made it possible to have all the plants used in this study being clonally propagated from a single mother plant.

In detail, twelve 3×4 meters plots were selected from the same field and each of them were 3 meters apart. Then, these plots were assigned to be as control (CK) and treatment with warming, N addition and warming+N addition stress, respectively. For warming treatment, these plants were warmed continuously from 2006 to 2009 with 200 cm×15 cm MSR-2420 infrared radiators (Kalglo Electronics Inc., Bethlehem, PA, USA). To avoid the influence of infrared radiators on nearby plots, the specially designed reflectors behind the heating elements were used to ensure both a nearly uniform irradiation of heated plots and confinement of the heating flux to such plots. In each unwarmed control subplot, there was one ‘dummy’ heater with the same shape and size as the infrared radiator, suspended with the same height, to simulate the shading effects of the heater. In manipulated warming experiments, infrared radiator is now widely used to increase temperature of warming plots, and there have been a large number of papers published in top journals [32]–[35]. With infrared radiators, the wavelength of heater radiation is in the range 800–1100 nm, hence the heaters produce negligible visible light or photosynthetically active radiation [36]. The N addition stress was sprayed with aqueous NH_4_NO_3_ (10 g N m^−2^) in mid-May every year. Within each plot, three individuals were randomly selected for further analysis and all of these samples were gathered in 2009. Then, a total of 36 individuals were collected in this study.

### DNA Extraction and Molecular Marker Analyses

Fresh leaf tissue was collected from each study individual and dried by silica gel. Total DNA was extracted using the hexadecyl-trimethyl-ammonium-bromide procedure [37]. For each individual, the same DNA sample was used as the starting material for the AFLP, MSAP and SSAP analyses described later.

The AFLP fingerprinting was exactly as described in Vos et al. [38], with minor modifications [39]. The MSAP protocol was performed according to Dong et al. [40]. For the SSAP procedure, genomic DNA was completely restricted with *Hpa*-II and *Msp*-I restriction enzyme (New England Biolabs, Massachusetts, USA), and simultaneously ligated to *Hpa*-II/*Msp*-I adapters (5′-GATCATGAGTCCTGCT-3′ and 5′-CGAGCAGGACTCATGA-3′). The restricted-ligated products were then pre-amplified with the *Hpa*-II/*Msp*-I primer (5′-ATCATGAGTCCTGCTCGG-3′) and retrotransponson primer (Bare-1∶5′-CTAGGGCATAATTCCAACAA-3′). For selective amplification, the pre-amplified products were diluted 20-fold and amplified with Bare-1 and *Hpa*-II/*Msp*-I selective primers (Table S1). All of the selective primer pairs of AFLP, MSAP and SSAP were listed in the Table S1.

For all markers, the selective amplified products were separated on 6% denaturing polyacrylamide gels with silver staining, and only clear and completely reproducible bands were scored.

### Statistical Analyses

The presence/absence of AFLP bands were determined by visual inspection and scored as 1/0. Similarly, the bands of MSAP and SSAP were first scored for presence (1) or absence (0) of *EcoR*-I/*Msp*-I and *EcoR*-I/*Hpa*-II fragments. To confirm the genetic similarity of the CK and other three treatment groups, we calculated the percentage of polymorphic loci based on the AFLP matrix. In addition, the changes of cytosine methylation levels and patterns were also estimated according to the MSAP and SSAP datasets. Then, the cytosine methylation alterations, which change their methylation status in treatment groups contrasting to CK, were divided into four types, CG hypo, CHG hypo, CG hyper and CHG hyper as described by Qi et al. [41]. In detail, the *Hpa*-II and *Msp*-I are two isoschizomers which recognize the same restriction site (5′-CCGG) but with differential sensitivity to methylation modifications of either C: *Hpa*-II can not cleave if either of the C is methylated, whereas *Msp*-I can not cleave if the external C is methylated. Therefore, for a given genotype, methylation of the internal or external C at the 5′-CCGG sites can be distinguished in the *EcoR*-I+*Hpa*-II/*Msp*-I-based MSAP fingerprinting profiles. So we hereby define these two major types of cytosine methylation at the 5′-CCGG sites as CG methylation (a band present in *Msp*-I-digest but absent from *Hpa*-II-digest) and CHG methylation (a band present in *Hpa*-II-digest but absent from *Msp*-I-digest), respectively. Accordingly, four patterns of methylation types, namely, CG hypo, CHG hypo, CG hyper and CHG hyper, could be defined. Then, the percentages of the four cytosine methylation types in each of the treatment group were scored. Meanwhile, in order to distinguish the specific loci methylation alterations from the random methylation variations, we also analyzed the percentages of differentially methylated loci (DML) that are polymorphic in CK but show monomorphism in treatment groups. Because if a treatment causing targeted methylation changes at specific loci, there should occur consistently in different sibling plants within the same treatment group [26]. To avoid the bias in parameter estimation, any loci present/absent in all but one individual were removed from the datasets. Thereafter, these DML of MSAP were excised from the gel and sequenced on an ABI 3730 automatic DNA analyzer (Applied Biosystems, California, USA). In addition, the bands absent in all of individuals in CK but showed monomorphic in treatment groups were also scored and sequenced. The procedure of isolated bands was performed as described by Sha et al. [42], and sequences homology searches were performed using the BLAST program (http://blast.ncbi.nlm.nih.gov/Blast.cgi). Furthermore, analysis of molecular variance (AMOVA) was employed to apportion the variation both within and between the four groups.

## Results

### Genetic Background of Control and Treatment Groups

**Figure 1:**
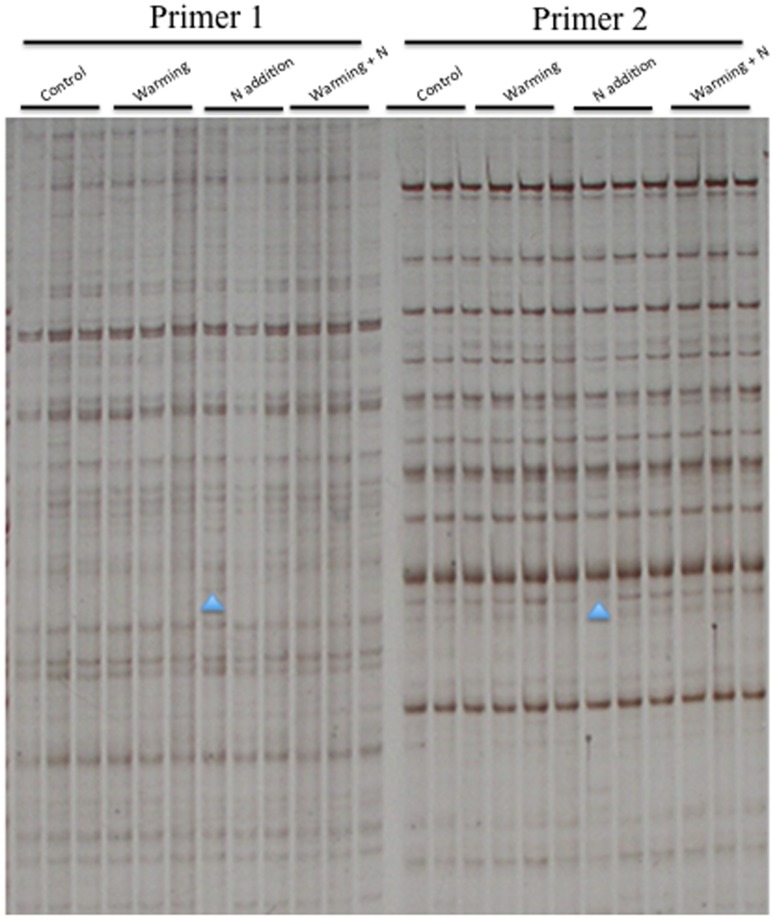
Examples of AFLP analysis on the genetic similarity of control and treatment groups. Possible variations are marked by arrowheads. (Primer 1: E+AAC/M+CAT; Primer 2: E+AAC/M+CTC).

Although the *L. chinensis* plant populations we used in this study were clonal and all derived from a single mother plant, both original genetic heterogeneity and newly arisen genetic changes may occur and may interwoven with the stress-induced epigenetic alterations that we aimed to stress. Thus, we first used the genetic marker AFLP to assess the genetic similarity of control and treatment groups. According to the results of AFLP, 14 primer combinations produced a total of 896 bands, with an average of 59.7 bands per primer pair (Figure 1 and Table S1). As expected, only 11 loci (represent 1.2% of the total bands) showed polymorphic bands across the entire dataset. The AFLP results indicated that there was no obvious genetic differentiation among the four groups. This result enables us to presume that all of these materials used in this study lacked appreciable genetic heterogeneity and the levels and patterns of methylation alterations to be detected should represent pure epigenetic variations.

### Levels and Patterns of Methylation Alteration in the Treatment Groups

**Figure 2:**
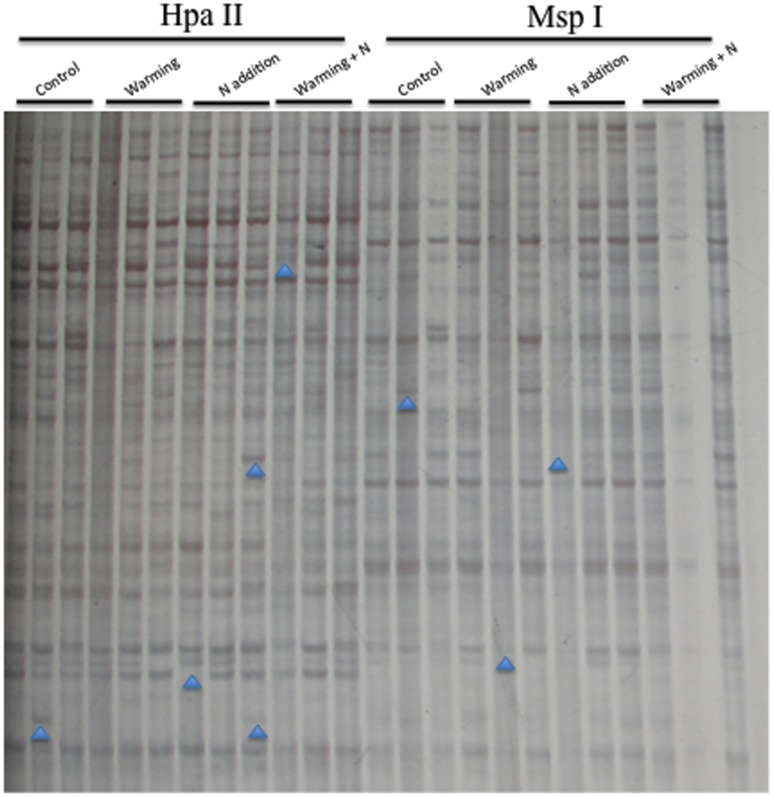
Examples of MSAP analysis on the control and treatment groups. Typical variations are marked by arrowheads. (Primer: E+AAC/H/M+TGC).

**Figure 3:**
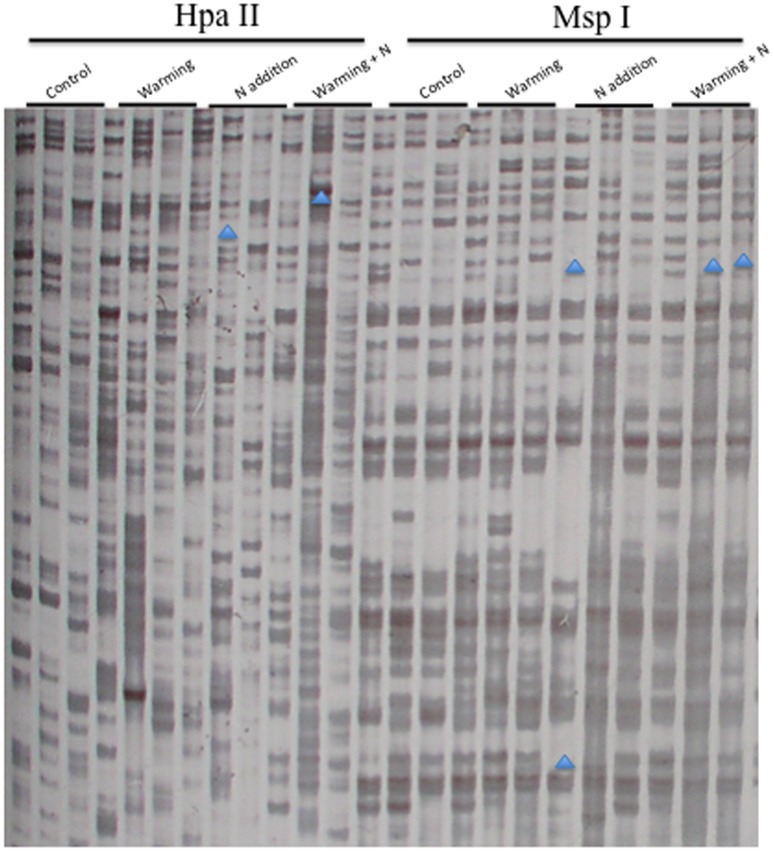
Examples of SSAP analysis on the control and treatment groups. Typical variations are marked by arrowheads. (Primer: BARE-1/H/M+TAG).

**Figure 4:**
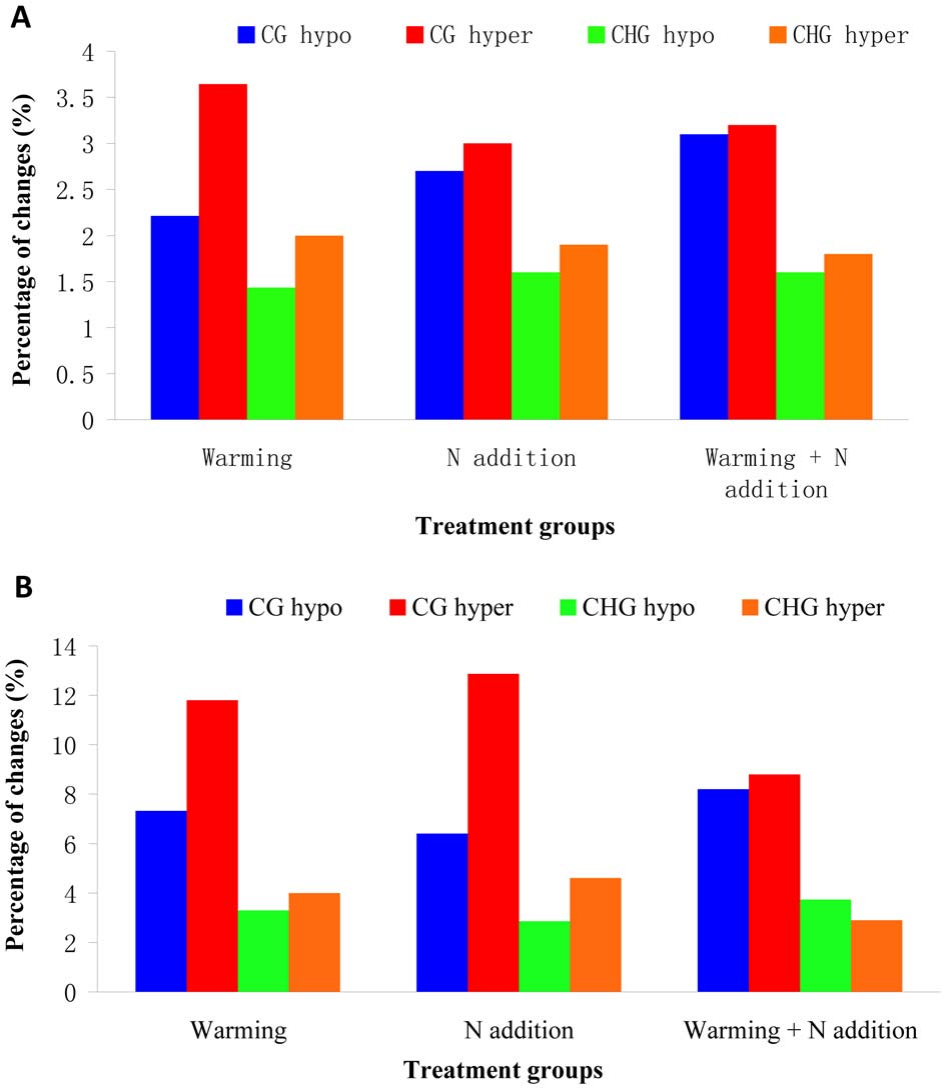
Tabulated changes of MSAP (A) and SSAP (B) profiles showing the four patterns of cytosine methylation alterations, CG hypo, CHG hypo, CG hyper and CHG hyper, in the warming, N addition and warming+N addition compared with the CK.

**Figure 5:**
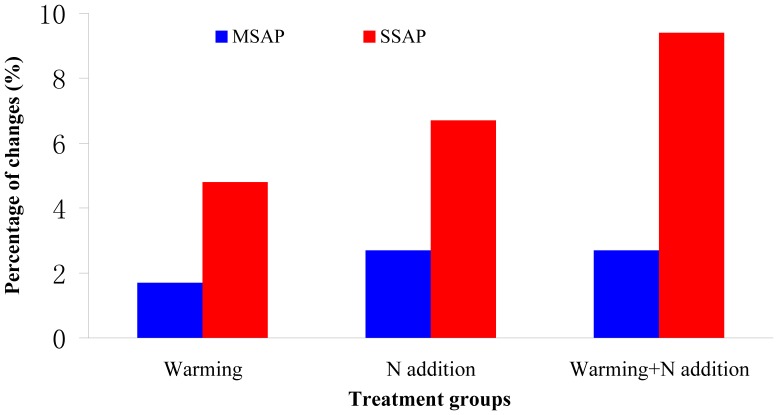
Tabulated differentially methylated loci (DML) in the three different treatment groups of *Leymus chinensis*, reveal by MSAP and SSAP. The percentages are calculated according to the variation patterns which are polymorphism in CK but exhibit monomorphism in the stress group.

To assess the levels and patterns of methylation alteration in the treatment groups, the MSAP and SSAP techniques were employed in this study. The data showed that, the 15 primer combinations assayed in the MSAP generated a total of 814 bands, with an average of 54.3 bands per primer pair (Figure 2 and Table S1). For SSAP analysis, a total of 642 bands were obtained from the 10 primer combinations, with an average of 64.2 bands per primer pair (Figure 3 and Table S1). These markers enabled us to assess the stress-associated epigenetic changes in the four types of cytosine methylation alterations (CG hypo, CHG hypo, CG hyper and CHG hyper). The results (Figure 4) indicated that, although the three treatment groups had similar frequencies of methylation variation patterns, the types of CG hyper and CHG hyper are higher than the CG hypo and CHG hypo in most of these comparisons. This indicated that the treatment groups have hypermethylated genomes than the untreated control. In addition, the percentages of all four patterns (CG hypo, CHG hypo, CG hyper and CHG hyper) in SSAP analysis were obviously higher than those in MSAP. This result indicated that the regions around the transposable elements had higher frequencies of cytosine methylation changes than other regions of the genome. Furthermore, comparing the percentages of the differentially methylated loci (DML) revealed that the warming+N addition group had the highest proportion of DML and the warming group the least (Figure 5). Similarly, we also found that the percentages of DML in SSAP apparently exceeded those in MSAP, suggesting higher rates of cytosine methylation alterations adjacent to the transposable elements.

To evaluate whether the treatments caused epigenetic differentiation, AMOVA was performed in this study based on both the MSAP and SSAP datasets (Table 1). As the MSAP marker revealed, 19.1% of the total variance presented among the four groups, it implies that the four groups had obvious differences in cytosine methylation patterns (F_ST_ = 0.19). In contrast, only a small amount of variation (6.76%) occurred among the four groups in SSAP analysis, suggesting that the regions around the transposable elements of the four groups have diverged only slightly in cytosine methylation variation patterns (F_ST_ = 0.07).

**Table 1:** Analysis of molecular variance (AMOVA) and F-statistics generated from the SSAP and MSAP datasets for the CK and treatment groups of *Leymus chinensis.*

	SSAP				MSAP			
Source of variation	d.f.	Sum ofsquares	Variancecomponents	Percentage ofvariation	d.f.	Sum ofsquares	Variancecomponents	Percentage ofvariation
Among groups	3	230.42	4.57	6.76	3	96.50	4.44	19.09
Within groups	8	504.67	63.08	93.24	8	150.67	18.83	80.91
Total	11	735.08	67.66		11	247.17	23.28	
Fixation index	F_ST_ = 0.07				F_ST_ = 0.19			

### Sequence Analysis of Differently Methylated Loci

Thirty-six differentially methylated DNA fragments of MSAP were recovered, cloned and sequenced, of which, 7 fragments showed significant homology to known sequences or genes (Table 2). Specifically, fragments F1, F21 and F22 were homologous to genomic sequences of *Oryza sativa*. F5, F16 and F20 showed similarity to *Hordeum vulgare* subsp. *vulgare* and F19 shared homology with sequences of *Triticum aestivum*. Additionally, fragments F1, F19 and F20 were mapped to the upstream and downstream of beta-expansin 1a precursor, glycosyltransferase and ethylene responsive transcription factor, respectively. The other fragments located on the gene body regions of tubby-like F-box protein, UNR-interacting protein, gag-pol polyprotein and DUF295 family protein, respectively.

**Table 2:** Sequence analysis of differentially methylated fragments isolated from MSAP in the stress groups of *Leymus chinensis*.

Fragment	Size (bp)	Location of sequences	Sequence homology	Species	Expect value	Genbank number
F1	131	5′ regulatory region	beta-expansin 1a precursor	*Oryza sativa*	2e–26	JQ231235
F5	268	5′ UTR	tubby-like F-box protein	*Hordeum vulgare* subsp. *vulgare*	4e–45	JQ231236
F16	401	5′ coding region	UNR-interacting protein	*Hordeum vulgare* subsp. *vulgare*	2e–57	JQ231237
F19	419	5′ regulatory region	glycosyltransferase	*Triticum aestivum*	1e–73	JQ231238
F20	336	3′ regulatory region	ethylene-responsive transcription factor	*Hordeum vulgare* subsp. *vulgare*	1e–26	JQ231239
F21	358	5′ coding region	gag-pol polyprotein	*Oryza sativa*	2e–19	JQ231240
F22	361	3′ coding region	DUF295 family protein	*Oryza sativa*	7e–56	JQ231241

## Discussion

Investigations of the effects of climate change on the terrestrial ecosystem have been discussed extensively during the past decades. A larger number of studies have illustrated that the changes in global environments have already not only caused distribution shift in plants and animals, but also have profound influences on the behavioral and morphological traits of species [8], [43]–[45]. For example, Pounds et al. [46] have demonstrated that 67% of the 110 or so species of *Atelopus* are likely to extinction and the large scale warming is a key factor in the disappearances. This conclusion is also supposed by Morin et al. [47] where have illustrated that the climate change has caused 16 North American tree species shift their distribution ranges at a continental scale. These previous studies have increased our understanding of how climate changes influence the terrestrial ecosystem.

In recent years, the use of genomic techniques to address how species adjust their genetic constitution to adapt to the changing environments constitutes an emerging fundamental issue. For instance, Manel et al. [48]–[50] illustrated that changes in temperature and precipitation conditions can caused the adaptive genetic variation in alpine plants at broad scale. Meanwhile, an array of studies have also implicated that heritable epigenetic variation could directly or indirectly contribute to the abilities of species to cope with changing environmental conditions [20], [28], [51]–[57]. These studies not only revealed the molecular mechanisms of plants in response and ultimate adaptation to changing environmental conditions, but also supplied a wealth of information towards our understanding of the evolutionary roles of epigenetic mechanisms.

In the present study, we have used multiple molecular markers including AFLP, MSAP and SSAP to investigate how natural populations of the perennial grass species *L. chinensis* alters its cytosine methylation status in response to warming, N addition and warming+N addition stresses. We presume that the warming factor used in this study has similar effects on plants as global warming which caused by human activities. Also, the influences of N addition on plants are similar to these of nitrogen deposition which caused by agricultural production. The purpose of this study is to demonstrate how and to what extent these factors induce the alterations in cytosine methylation modification. Given the theoretical expectations and empirical results, epigenetic variations may usually be affected by the genetic composition. Therefore, it is difficult to distinguish the epigenetic from genetic variations in genetically diverse individuals and populations [26], [58]. In considering the effects of genetic variations, we used plants all propagated asexually from a single mother plant. In addition, we used the AFLP marker to assess the genetic similarity of both the control and treatment groups. As revealed in our results, although the materials used in this study were treated with abiotic stresses for four consecutive generations, only 1.2% of a total of 896 bands showed genetic polymorphism, indicating that all the samples used in this study shared the same genetic heritage. As such, the alterations of cytosine methylation status of each individual retrieved from MSAP should be independent of genetic variations.

The observation described above enables us to evaluate the warming, N addition and warming+N addition stress-induced changes in the species *L. chinensis*. By analyzing the hyper- and hypomethylation of CG and CHG, we found that all the three treatment groups showed alterations in cytosine methylation pattern. Similar results were also found in some previous studies which illustrated that abiotic stresses could alter the cytosine methylation status through hypo- and hypermethylation of DNA [59]–[63]. Together, our findings suggested that the changes of cytosine methylation states of each treatment group might be induced by the warming and N addition. Interestingly, in our comparisons we noted that the levels of cytosine methylation alteration around the transposable elements were obviously higher than the other genomic regions. Activation of transposable elements in response and subsequent adaptation to stress has been documented previously [64], [65]. Also, several studies have illustrated that the abiotic stresses, including drought, temperature and nutrient-deficiency, could activate the transposable elements as a result of altered cytosine methylation states [66]–[73]. However, transposable elements are highly unstable and stress treatments may induce both genetic and epigenetic variations. To distinguish the epigenetic from genetic variations, we applied the AFLP technique to analyses the genetic structures of both the CK and treatment groups, and our results illustrated that all the samples used in this study shared a similar genetic background. Therefore, we assume that most of these variations detected by SSAP were retrieved from epigenetic variation. Accordingly, our results have not only verified the previous findings, but also further demonstrated that the different genomic regions of *L. chinensis* showed differential propensity for cytosine methylation alterations.

As illustrated by our experiment and some previous studies, that the status of cytosine methylation could be modified by abiotic stresses, and some of these induced changes are faithfully transmitted to offspring [26]. More recently, however, several studies in *Arabidopsis* demonstrated that the alterations in cytosine methylation modification could occur spontaneously without the pressures of stress, and some of these transgenerational cytosine methylation changes could also generate new allelic states that underlie changes in gene transcription, and hence, providing a novel mechanism for phenotypic variation [74], [75]. Therefore, in this study, to confirm whether the cytosine methylation alterations were triggered by abiotic stress and distinguish the targeted methylation changes at specific loci from random methylation alterations, the percentages of DML in SSAP and MSAP of each treatment group were scored. As revealed by our analysis, all of these treatment groups, including DML and the percentages of SSAP, were obviously higher than those in MSAP (Fig. 4). These results are concordant with the hyper- and hypomethylation analyses in this study. In general, our findings are in agreement with the study of *Taraxacum officinale* that the cytosine methylation patterns showed high specificity between the control and treatment groups [26]. Furthermore, according to the gene function analysis, some DML showed significant homologies to known sequences or genes (Table 2). Take the case of fragment F5, for example, it shared homology with the tubby-like F-box family protein which may participate in the abscisic acid (ABA) signaling pathway [76]. In addition, the fragment F20 was homologous to the 3′ regulatory region of the ethylene-responsive transcription factor. Both of these genes could contribute to the abilities of plants to cope with abiotic stresses [77]. Taken together, our findings implied that these DML might play important roles in adaptation by the stressed plants to the particular abiotic stress conditions.

The aforesaid analyses illustrated that the warming, N addition and warming+N addition stresses had a clear impact on the cytosine methylation status of the stressed *L. chinensis* plants. However, whether the abiotic stresses could trigger specific methylation changes and then lead to epigenetic differentiation between the control and treatment populations remained to be addressed. However, it is notable that in comparing the cytosine methylation patterns, we found that the control and treatment groups indeed showed epigenetic differentiation (F_ST_ = 0.19 for MSAP and F_ST_ = 0.07 for SSAP). Variation in methylation levels and patterns among natural populations has been reported in several previous studies [20], [28], [78], [79]. These studies revealed that the cytosine methylation status could be triggered by exposure to different environmental conditions, and then lead to rapid epigenetic differentiation. Nonetheless, natural populations inherently harbor genetic variations, and hence, making the single out of epigenetic variations difficult. As illustrated in our analysis, all plants used in this study, being derived from a single mother plant via asexual propagation, shared nearly the same genetic heritage, and hence, the observed epigenetic variations were not associated with genetic variations. It is therefore possible that these pure epigenetic differentiations were occurred *de novo* as a result of the abiotic stresses.

In conclusion, our findings have clearly shown that warming, N addition and warming+N addition stresses readily induced cytosine methylation changes and the extent of which was variable across different genomic regions. It is reasonable to assume that this flexibility of stress-induced epigenetic modifications are consequential to enhanced adaptation by the stressed plants, and which may bear relevance to global warming and nitrogen deposition.

## Supporting Information

Table S1 **The selective amplification primer pairs of the amplified fragment length polymorphism (AFLP), methylation-sensitive amplified polymorphism (MSAP) and retrotransposon based sequence-specific amplification polymorphism (SSAP) used in this study.** NB represents the number of bands.
(DOCX)

## References

[1] Hughes L (2000) Biological consequences of global warming: is the signal already. Trends in Ecology & Evolution 15: 56–61. 10652556 10.1016/s0169-5347(99)01764-4

[2] Peter MV, Harold AM, Jane L, Jerry MM (1997) Human domination of earth’s ecosystems. Science 277: 494–499.

[3] Thomas RK, Kevin ET (2003) Modern global climate change. Science 302: 1719. 14657489 10.1126/science.1090228

[4] Peter MV (1994) Beyond global warming: ecology and global change. Ecology 75: 1861–1876.

[5] Vitousek PM, Aber JD, Howarth RW, Likens GE, Matson PA, et al (1997) Human alteration of the global nitrogen cycle: sources and consequences. Ecological Applications 7: 737–750.

[6] Duce RA, LaRoche J, Altieri K, Arrigo KR, Baker AR, et al (2008) Impacts of atmospheric anthropogenic nitrogen on the open ocean. Science 320: 893–897. 18487184 10.1126/science.1150369

[7] Bouwman AF, Beusen AHW, Billen G (2009) Human alteration of the global nitrogen and phosphorus soil balances for the period 1970–2050. Global Biogeochemical Cycles 23: GB0A04 (doi:10.1029/2009GB003576)..

[8] Lovejoy TE, Hannah L (2004) Climate Change and Biodiversity. New Haven: Yale University Press. 440 p.

[9] Bijlsma R, Loeschcke V (2005) Environmental stress, adaptation and evolution: an overview. Journal of Evolutionary Biology 18: 744–749. 16033544 10.1111/j.1420-9101.2005.00962.x

[10] Lang-Mladek C, Popova O, Kiok K, Berlinger M, Rakic B, et al (2010) Transgenerational inheritance and resetting of stress-induced loss of epigenetic gene silencing in *Arabidopsis* . Molecular Plant 3: 594–602. 20410255 10.1093/mp/ssq014 PMC2877484

[11] Boyko A, Kovalchuk I (2011) Genome instability and epigenetic modification–heritable responses to environmental stress? Current Opinion in Plant Biology 14: 260–266. 21440490 10.1016/j.pbi.2011.03.003

[12] Savolainen O, Bokma F, Garcia-Gil R, Komulainen P, Repo T (2004) Genetic variation in cessation of growth and frost hardiness and consequences for adaptation of *Pinus sylvestris* to climatic changes. Forest Ecology and Management 197: 79–89.

[13] Davis MB, Shaw RG, Etterson JR (2005) Evolutionary responses to changing climate. Ecology 86: 1704–1714.

[14] Manel S, Poncet BN, Legendre P, Gugerli F, Holderegger R (2010) Common factors drive adaptive genetic variation at different spatial scales in *Arabis alpina* . Molecular Ecology 19: 3824–3835. 20723057 10.1111/j.1365-294X.2010.04716.x

[15] Katori T, Ikeda A, Iuchi S, Kobayashi M, Shinozaki K, et al (2010) Dissecting the genetic control of natural variation in salt tolerance of *Arabidopsis thaliana* accessions. The Journal of Experimental Botany 61: 1125–1138. 20080827 10.1093/jxb/erp376 PMC2826654

[16] Yakovlev IA, Asante DK, Fossdal CG, Junttila O, Johnsen O (2011) Differential gene expression related to an epigenetic memory affecting climatic adaptation in Norway spruce. Plant Science 180: 132–139. 21421355 10.1016/j.plantsci.2010.07.004

[17] Trontin C, Tisne S, Bach L, Loudet O (2011) What does *Arabidopsis* natural variation teach us (and does not teach us) about adaptation in plants? Current Opinion in Plant Biology 14: 225–231. 21536479 10.1016/j.pbi.2011.03.024

[18] Jablonka E, Raz G (2009) Transgenerational epigenetic inheritance: Prevalence, mechanisms, and implications for the study of heredity and evolution. The Quarterly Review of Biology 84: 131–176. 19606595 10.1086/598822

[19] Keyte AL, Percifield R, Liu B, Wendel JF (2006) Intraspecific DNA methylation polymorphism in cotton (*Gossypium hirsutum* L.). Journal of Heredity 97: 444–450. 16987937 10.1093/jhered/esl023

[20] Lira-Medeiros CF, Parisod C, Fernandes RA, Mata CS, Cardoso MA, et al (2010) Epigenetic variation in mangrove plants occurring in contrasting natural environment. PloS ONE 5: e10326. 20436669 10.1371/journal.pone.0010326 PMC2859934

[21] Rapp RA, Wendel JF (2005) Epigenetics and plant evolution. New Phytologist 168: 81–91. 16159323 10.1111/j.1469-8137.2005.01491.x

[22] Kim JM, To TK, Nishioka T, Seki M (2010) Chromatin regulation functions in plant abiotic stress responses. Plant Cell and Environment 33: 604–611. 10.1111/j.1365-3040.2009.02076.x 19930132

[23] Yamaguchi-Shinozaki K, Shinozaki K (2006) Transcriptional regulatory networks in cellular responses and tolerance to dehydration and cold stresses. Annual Review of Plant Biology 57: 781–803. 10.1146/annurev.arplant.57.032905.105444 16669782

[24] Lisch D (2009) Epigenetic regulation of transposable elements in plants. Annual Review of Plant Biology 60: 43–66. 10.1146/annurev.arplant.59.032607.092744 19007329

[25] Chinnusamy V, Zhu JK (2009) Epigenetic regulation of stress responses in plants. Current Opinion in Plant Biology 12: 133–139. 19179104 10.1016/j.pbi.2008.12.006 PMC3139470

[26] Verhoeven KJ, Jansen JJ, Dijk PJ, Biere A (2010) Stress-induced DNA methylation changes and their heritability in asexual dandelions. New Phytologist 185: 1108–1118. 20003072 10.1111/j.1469-8137.2009.03121.x

[27] Boyko A, Kathiria P, Zemp FJ, Yao Y, Pogribny I, et al (2007) Transgenerational changes in the genome stability and methylation in pathogen-infected plants (Virus-induced plant genome instability). Nucleic Acids Research 35: 1714–1725. 17311811 10.1093/nar/gkm029 PMC1865051

[28] Herrera CM, Bazaga P (2011) Untangling individual variation in natural populations: ecological, genetic and epigenetic correlates of long-term inequality in herbivory. Molecular Ecology 20: 1675–1688. 21466603 10.1111/j.1365-294X.2011.05026.x

[29] Roy SJ, Tucker EJ, Tester M (2011) Genetic analysis of abiotic stress tolerance in crops. Current Opinion in Plant Biology 14: 232–239. 21478049 10.1016/j.pbi.2011.03.002

[30] Zhu TC (2004) Biological Ecology of *Leymus chinensis*. Changchun: Jilin Science & Technology. 523–609.

[31] Gao Y, Wang D, Ba L, Bai Y, Liu B (2008) Interactions between herbivory and resource availability on grazing tolerance of *Leymus chinensis* . Environmental and Experimental Botany 63: 113–122.

[32] Luo Y, Wan S, Hui D, Wallace LL (2001) Acclimatization of soil respiration to warming in a tall grass prairie. Nature 413: 622–625. 11675783 10.1038/35098065

[33] Niu S, Wu M, Han Y, Xia J, Li L, et al (2008) Water-mediated responses of ecosystem carbon fluxes to climatic change in a temperate steppe. New Phytologist 177: 209–219. 17944829 10.1111/j.1469-8137.2007.02237.x

[34] Niu S, Xing X, Zhang Z, Xia J, Zhou X, et al (2011) Water-use efficiency in response to climate change: From leaf to ecosystem in a temperate steppe. Global Change Biology 17: 1073–1082.

[35] Wan S, Xia J, Liu W, Niu S (2009) Photosynthetic overcompensation under nocturnal warming enhances grassland carbon sequestration. Ecology 90: 2700–2710. 19886480 10.1890/08-2026.1

[36] Saleska SR, Harte J, Torn MS (1999) The effect of experimental ecosystem warming on CO_2_ fluxes in a montane meadow. Global Change Biology 5: 125–141.

[37] Doyle JJ, Doyle JL (1987) A rapid DNA isolation procedure for small quantities of leaf tissue. Phytochemical Bulletin 19: 11–15.

[38] Vos P, Hogers R, Bleeker M, Reijans M, Lee T, et al (1995) AFLP: a new technique for DNA fingerprinting. Nucleic Acids Research 23: 4407–4414. 7501463 10.1093/nar/23.21.4407 PMC307397

[39] Wang YM, Dong ZY, Zhang ZJ, Lin XY, Shen Y, et al (2005) Extensive de novo genomic variation in rice induced by introgression from wild rice (*Zizania latifolia* Griseb). Genetics 170: 1945–1956. 15937131 10.1534/genetics.105.040964 PMC1449789

[40] Dong ZY, Wang YM, Zhang ZJ, Shen Y, Lin XY, et al (2006) Extent and pattern of DNA methylation alteration in rice lines derived from introgressive hybridization of rice and *Zizania latifolia* Griseb. Theoretical and Applied Genetics 113: 196–205. 16791687 10.1007/s00122-006-0286-2

[41] Qi B, Zhong X, Zhu B, Zhao N, Xu L, et al (2010) Generality and characteristics of genetic and epigenetic changes in newly synthesized allotetraploid wheat lines. Journal of Genetics and Genomics 37: 737–748. 21115168 10.1016/S1673-8527(09)60091-6

[42] Sha AH, Lin XH, Huang JB, Zhang DP (2005) Analysis of DNA methylation related to rice adult plant resistance to bacterial blight based on methylation-sensitive AFLP (MSAP) analysis. Molecular Genetics and Genomics 273: 484–490. 15968537 10.1007/s00438-005-1148-3

[43] Parmesan C (2006) Ecological and evolutionary responses to recent climate change. Annual Review of Ecology, Evolution, and Systematics 37: 637–669.

[44] Reusch TBH, Wood TE (2007) Molecular ecology of global change. Molecular Ecology 16: 3973–3992. 17894755 10.1111/j.1365-294X.2007.03454.x

[45] Mawdsley JR, O’Malley R, Ojima DS (2009) A Review of Climate-Change Adaptation Strategies for Wildlife Management and Biodiversity Conservation. Conservation Biology 23: 1080–1089. 19549219 10.1111/j.1523-1739.2009.01264.x

[46] Pounds JA, Bustamante MR, Coloma LA, Consuegra JA, Fogden MPL, et al (2006) Widespread amphibian extinctions from epidemic disease driven by global warming. Nature 439: 161–167. 16407945 10.1038/nature04246

[47] Morin X, Viner D, Chunine I (2008) Tree species range shifts at a continental scale: new predictive insights from a process-based model. Journal of Ecology 96: 784–794.

[48] Manel S, Poncet BN, Legendre P, Gugerli F, Holderegger R (2010a) Common factors drive adaptive genetic variation at different spatial scales in *Arabis alpina* . Molecular Ecology 19: 3824–3835. 20723057 10.1111/j.1365-294X.2010.04716.x

[49] Manel S, Joost S, Epperson B (2010b) Perspectives o the use of landscape genetic to detect genetic adaptive variation in the field. Molecular Ecology 19: 3760–3772. 20723056 10.1111/j.1365-294X.2010.04717.x

[50] Manel S, Gugerli F, Thuiller W, Alvarez N, Legendre P, et al (2012) Broad-Scale adaptive genetic variation in alpine plants is driven by temperature and precipitation. Molecualr Ecology 21: 3729–3738. 10.1111/j.1365-294X.2012.05656.x PMC4003392 22680783

[51] Huang NC, Li CH, Lee JY, Yen HE (2010) Cytosine methylation changes in the ice plant *Ppc1* promoter during transition from C3 to Crassulacean acid metabolism. Plant Science 178: 41–46.

[52] Paun O, Bateman RM, Fay MF, Hedren M, Civeyrel L, et al (2010) Stable epigenetic effects impact adaptation in allopolyploid orchids (*Dactylorhiza*: Orchidaceae). Molecular Biology and Evolution 27: 2465–2473. 20551043 10.1093/molbev/msq150 PMC2955735

[53] Richards EJ (2011) Natural epigenetic variation in plant species a view from the field: a view from the field. Current Opinion in Plant Biology 14: 204–209. 21478048 10.1016/j.pbi.2011.03.009

[54] Grativol C, Hemerly AS, Ferreira PC (2011) Genetic and epigenetic regulation of stress responses in natural plant populations. Biochimica et Biophysica Acta 1819: 176–185. 21914492 10.1016/j.bbagrm.2011.08.010

[55] Kalisz S, Purugganan MD (2004) Epialleles via DNA methylation: consequences for plant evolution. Trends in Ecology and Evolution 19: 309–314. 16701276 10.1016/j.tree.2004.03.034

[56] Rapp RA, Wendel JF (2005) Epigenetics and plant evolution. New Phytologist 168: 81–91. 16159323 10.1111/j.1469-8137.2005.01491.x

[57] Jablonka E, Raz G (2009) Transgenerational epigenetic inheritance: prevalence, mechanisms, and implications for the study of heredity and evolution. Quarterly Review of Biology 84: 131–176. 19606595 10.1086/598822

[58] Johannes F, Porcher E, Teixeira FK, Saliba-Colombani V, Simon M, et al (2009) Assessing the impact of transgenerational epigenetic variation on complex traits. PLoS Genetics 5: e1000530. 19557164 10.1371/journal.pgen.1000530 PMC2696037

[59] Kovarik A, Koukalova B, Bezdek M, Opatrny Z (1997) Hypermethylation of tobacco heterochromatic loci in response to osmotic stress. Theoretical and Applied Genetics 95: 301–306.

[60] Choi CS, Sano H (2007) Abiotic-stress induces demethylation and transcriptional activation of a gene encoding a glycerophosphodiesterase-like protein in tobacco plants. Molecular Genetics and Genomics 277: 589–600. 17273870 10.1007/s00438-007-0209-1

[61] Steward N, Ito M, Yamaguchi Y, Koizumi N, Sano H (2002) Periodic DNA methylation in maize nucleosomes and demethylation by environmental stress. The Journal of Biological Chemistry 277: 37741–37746. 12124387 10.1074/jbc.M204050200

[62] Santos AP, Ferreira L, Maroco J, Oliveira MM (2011) Abiotic stress and induced DNA hypomethylation cause interphase chromatin structural changes in rice rDNA loci. Gytogenetic and Genome Research 132: 297–303. 10.1159/000322287 21307636

[63] Yaish MW, Colasanti J, Rothstein SJ (2011) The role of epigenetic processes in controlling flowering time in plants exposed to stress. Journal of Experimental Botany 62: 3727–3735. 21633082 10.1093/jxb/err177

[64] McClintock B (1984) The significance of responses of the genome to challenge. Science 226: 792–801. 15739260 10.1126/science.15739260

[65] Boyko A, Kovalchuk I (2008) Epigenetic control of plant stress response. Environmental and Molecular Mutagenesis 49: 61–72. 17948278 10.1002/em.20347

[66] Zhang XY (2008) The epigenetic landscape of plants. Science 320: 489–492. 18436779 10.1126/science.1153996

[67] Kalendar R, Tanskanen J, Immonen S, Nevo E, Schulman AH (2000) Genome evolution of wild barley (*Hordeum spontaneum*) by BARE-1 retrotransposon dynamics in response to sharp microclimatic divergence. Proceedings of the National Academy of Sciences 97: 6603–6607. 10.1073/pnas.110587497 PMC18673 10823912

[68] Beguiristain T, Grandbastien MA, Puigdomenech P, Casacuberta M (2001) Three Tnt1 subfamilies show different stress-associated pattern of expression in tobacco. Consequences for retrotransposon control and evolution in plants. Plant Physiology 127: 212–221. 11553749 10.1104/pp.127.1.212 PMC117977

[69] Jiang N, Bao Z, Zhang X, Hirochika H, Eddy SR, et al (2003) An active DNA transposon family in rice. Nature 421: 163. 12520302 10.1038/nature01214

[70] Hashida SN, Uchiyama T, Martin C, Kishima Y, Sano Y, et al (2006) The temperature-dependent change in methylation of the *Antirrhinum transposon* Tam3 is controlled by the activity of its transposase. Plant Cell 18: 104–118. 16326924 10.1105/tpc.105.037655 PMC1323487

[71] Hashida SN, Kitamura K, Mikami T, Kishima Y (2003) Temperature shift coordinately changes the activity and the methylation state of transposon Tam3 in *Antirrhinum majus* . Plant Physiology 132: 1207. 12857803 10.1104/pp.102.017533 PMC167061

[72] Tsukahara S, Kobayashi A, Kawabe A, Mathieu O, Miura A, et al (2009) Bursts of retrotransposition reproduced in *Arabidopsis* . Nature 461: 423–426. 19734880 10.1038/nature08351

[73] Miura A, Yonebayashi S, Watanabe K, Toyama T, Shimada H, et al (2001) Mobilization of transposons by amutation abolishing full DNA methylation in *Arabidopsis* . Nature 411: 212–214. 11346800 10.1038/35075612

[74] Robert JS, Matthew DS, Mathew GL, Ronan CO, Mark AU, et al (2011) Transgenerational epigenetic instability is a source of novel methylation variants. Science 334: 369–373. 21921155 10.1126/science.1212959 PMC3210014

[75] Becker C, Hagmann J, Müller J, Koenig D, Stegle O, et al (2011) Spontaneous epigenetic variation in the *Arabidopsis thaliana* methylome. Nature 480: 245–249. 22057020 10.1038/nature10555

[76] Lai CP, Lee CL, Chen PH, Wu SH, Yang CC, et al (2004) Molecular analyses of the *Arabidopsis* TUBBY-like protein gene family. Plant Physiology 134: 1586–1597. 15064372 10.1104/pp.103.037820 PMC419833

[77] Ohta M, Ohme-Takagi M, Shinshi H (2000) Three ethylene-responsive transcription factors in tobacco with distinct transactivation functions. The Plant Journal 22: 29–38. 10792818 10.1046/j.1365-313x.2000.00709.x

[78] Herrera CM, Bazaga P (2010) Epigenetic differentiation and relationship to adaptive genetic divergence in discrete populations of the violet *Viola cazorlensis* . New Phytologist 187: 867–876. 20497347 10.1111/j.1469-8137.2010.03298.x

[79] Li Y, Shan X, Liu X, Hu L, Guo W, et al (2008) Utility of the methylation-sensitive amplified polymorphism (MSAP) marker for detection of DNA methylation polymorphism and epigenetic population structure in a wild barley species (*Hordeum brevisubulatum*). Ecological Research 23: 927–930.

