# Supplementary material for: Cytosine Methylation Alteration in Natural Populations of Leymus chinensis Induced by Multiple Abiotic Stresses
Source: PLoS One. 2013 Feb 13;8(2):e55772. doi: 10.1371/journal.pone.0055772 (PMC3572093; doi:10.1371/journal.pone.0055772)
Supplement: Table S1 — The selective amplification primer pairs of the amplified fragment length polymorphism (AFLP), methylation-sensitive amplified polymorphism (MSAP) and retrotransposon based sequence-specific amplification polymorphism (SSAP) used in this study. NB represents the number of bands. (DOCX) [file pone.0055772.s001.docx]

**Supporting Information legend:**

**Table S1.** The selective amplification primer pairs of the amplified fragment length polymorphism (AFLP), methylation-sensitive amplified polymorphism (MSAP) and retrotransposon based sequence-specific amplification polymorphism (SSAP) used in this study. NB represents the number of bands.

| AFLP | NB | | MSAP | | NB | | SSAP | | NB | |  |
| --- | --- | --- | --- | --- | --- | --- | --- | --- | --- | --- | --- |
| E+AAC / M+CAG | 69 | | E+AAC / H/M+TGC | | 72 | | BARE-1 / H/M+TAC | | 62 | |  |
| E+AAC / M+CAT | 71 | | E+AAG / H/M+TAC | | 64 | | BARE-1 / H/M+TAG | | 103 | |  |
| E+AAC / M+CTC | 68 | | E+AAG / H/M+TAG | | 87 | | BARE-1 / H/M+TCT | | 78 | |  |
| E+AAC / M+CTG | 79 | | E+AAG / H/M+TCT | | 53 | | BARE-1 / H/M+TCG | | 44 | |  |
| E+AAC / M+CTT | 56 | | E+AAG / H/M+TCG | | 52 | | BARE-1 / H/M+TTC | | 58 | |  |
| E+AAG / M+CAA | 49 | | E+AAG / H/M+TTA | | 95 | | BARE-1 / H/M+TTG | | 76 | |  |
| E+AAG / M+CAT | 46 | | E+-ACA / H/M+TAC | | 41 | | BARE-1 / H/M+TTA | | 52 | |  |
| E+AAG / M+CTA | 61 | | E+ACA / H/M+TCG | | 27 | | BARE-1 / H/M+TGA | | 65 | |  |
| E+AAG / M+CTC | | 64 | | E+ACA / H/M+TGC | | 21 | | BARE-1 / H/M+TGC | | 42 | |
| E+AAG / M+CTG | 57 | | E+ACT / H/M+TTC | | 27 | | BARE-1 / H/M+TGT | | 62 | |  |
| E+ ACT / M+CAC | 54 | | E+ACT / H/M+TTA | | 25 | |  | |  | |  |
| E+ ACT / M+CAG | 66 | | E+ACT / H/M+TGC | | 69 | |  | |  | |  |
| E+ ACT / M+CAT | 58 | | E+ACC / H/M+TAC | | 70 | |  | |  | |  |
| E+ ACT / M+CTA | 45 | | E+ACC / H/M+TCT | | 58 | |  | |  | |  |
|  |  | | E+ACC / H/M+TGC | | 53 | |  | |  | |  |
